# Supplementary material for: Management of neonatal jaundice in low- and lower-middle-income countries
Source: BMJ Paediatr Open. 2019 Feb 14;3(1):e000408. doi: 10.1136/bmjpo-2018-000408 (PMC6422246; doi:10.1136/bmjpo-2018-000408)
Supplement: Author's manuscript [file bmjpo-2018-000408.draft_revisions.pdf]

BMJ Paediatrics Open

BMJ Paediatrics Open is committed to open peer review. As part of this commitment we make the peer review history of every article we publish publicly available.

When an article is published we post the peer reviewers' comments and the authors' responses online. We also post the versions of the paper that were used during peer review. These are the versions that the peer review comments apply to.

The versions of the paper that follow are the versions that were submitted during the peer review process. They are not the versions of record or the final published versions. They should not be cited or distributed as the published version of this manuscript.

BMJ Paediatrics Open is an open access journal and the full, final, typeset and author-corrected version of record of the manuscript is available on our site with no access controls, subscription charges or pay-per-view fees (http://bmjpaedsopen.bmj.com).

If you have any questions on BMJ Paediatrics Open's open peer review process please email info.bmjpo@bmj.com

BMJ Paediatrics Open

Management of neonatal jaundice in low and lower middle income countries

Journal:	*BMJ Paediatrics Open*
Manuscript ID	bmjpo-2018-000408
Article Type:	Editorial
Date Submitted by the Author:	04-Jan-2019
Complete List of Authors:	Mir, Shahryar; Dijklander Hospital, Paediatrics<br>van der Geest, Berthe; Erasmus MC, Paediatrics<br>Been, Jasper; Erasmus MC, Paediatrics
Keywords:	Comm Child Health, Epidemiology, Jaundice, Neonatology, Screening

SCHOLARONE™
Manuscripts

Management of neonatal jaundice in low and lower middle income countries

Shahryar E. Mir[1], Berthe A.M. van der Geest[2,3], Jasper V. Been[2-4*]

[1]Department of Paediatrics, Dijklander Hospital, Purmerend, the Netherlands

[2]Division of Neonatology, Department of Paediatrics, Erasmus MC – Sophia Children's Hospital, University Medical Centre Rotterdam, Rotterdam, the Netherlands

[3]Department of Obstetrics and Gynaecology, Erasmus MC, University Medical Centre Rotterdam, Rotterdam, the Netherlands

[4]Department of Public Health, Erasmus MC, University Medical Centre Rotterdam, Rotterdam, the Netherlands

[*]Corresponding author: Dr. Jasper V. Been, Division of Neonatology, Department of Paediatrics, Erasmus MC – Sophia Children's Hospital, University Medical Centre Rotterdam, PO Box 2060, 3000 CB Rotterdam, the Netherlands;

E: j.been@erasmusmc.nl; T: +31-10-7037048; F: +31-10-7036815.

Word count: 931

Confidential: For Review Only

More than 85% of newborns develop some degree of jaundice during the first days of life. Often a benign condition, the yellowish discolouration of the skin, sclerae, and mucous membranes in newborn infants is caused by unconjugated hyperbilirubinaemia, which may result from increased production of bilirubin, limited ability to conjugate bilirubin, and/or slow hepatic-enteric clearance of bilirubin.[1] A small proportion of newborns develops extreme hyperbilirubinaemia, i.e. a total serum bilirubin (TSB) level of 25 mg/dL (428 µmol/L) or more. When it is not timely recognised or treated, extreme unconjugated hyperbilirubinaemia may lead to acute bilirubin encephalopathy. This in turn carries a risk of developing a spectrum of long-term neurological sequelae known as kernicterus spectrum disorders (KSD), encompassing “classical kernicterus” but also milder forms of permanent brain damage caused by bilirubin neurotoxicity.[1,2] Worldwide, it is estimated that extreme hyperbilirubinaemia affects at least 481,000 late-preterm and term newborn infants annually, resulting in 114,000 deaths and more than 63,000 survivors with moderate or severe long-term disability.[1,3] More than 75% of affected infants live in low and lower middle-income countries. Moreover, in South Asia severe hyperbilirubinaemia is the seventh leading cause of neonatal mortality.[1]

When timely recognised, clinically significant hyperbilirubinaemia can be treated with conventional phototherapy and in more severe cases exchange transfusion, thus reducing the risk of KSD. Relatively uncommon in high-income countries, exchange transfusions are performed regularly in low and lower middle-income countries.[3] In these countries early recognition of hyperbilirubinaemia can be difficult. In low-resource settings home births are common, and for institutional deliveries early post-discharge follow-up controls are not always feasible. In addition, parental unawareness and logistical challenges may result in poor care-seeking behaviour. In these settings infants often present with extreme hyperbilirubinemia and moderate to severe stages of acute bilirubin encephalopathy, resulting in high mortality and morbidity rates.[1,3] Traditionally, neonatal jaundice is determined by visual inspection. If severe jaundice is suspected, TSB is quantified in blood to assess the need for treatment. However, visual inspection of neonatal jaundice correlates poorly with the TSB level, particularly in non-Caucasian infants.[1] Moreover, in areas with limited resources laboratory testing can be challenging due to the high cost and lack of trained technical personnel. Transcutaneous bilirubin (TcB) devices have been developed as potential non-invasive alternatives to visual inspection. Several observational studies have shown a good correlation between TSB and TcB measurements. TcB can be performed by nurses or trained health workers with limited education. Coordinated TcB screening in a Canadian community setting was associated with a 55% reduction in severe hyperbilirubinaemia compared with visual inspection alone.[4]

Confidential: For Review Only

Shah *et al.* have evaluated a care improvement initiative in a large hospital in Karachi, the largest city in Pakistan.[5] TcB screening of visibly jaundiced, otherwise well babies was implemented and clinical characteristics and jaundice-related outcomes were compared in the six months before versus the six months after implementation. There was a 79% reduction in the number of blood samples taken for TSB quantification following TcB implementation; at the same time the proportion of infants receiving phototherapy increased (6% after vs. 1% before implementation of TcB). Overall this is a useful uncontrolled before-after study, and the first to investigate the effectiveness of using a TcB device to diagnose neonatal jaundice in a Pakistani population. The considerable reduction in TSB sampling following TcB implementation as compared to other studies (in general reduction of 40 to 50%), was attributed to excessive sampling due to the high incidence of severe hyperbilirubinaemia (15%) in South Asia. The increased use of phototherapy in the post-implementation period suggests improved recognition of hyperbilirubinaemia, although it is possible that this includes some over-treatment. In this study the vast majority of infants were indeed screened, suggesting a low threshold for selection for screening. It is however important to note that selective screening based on visible jaundice carries a risk of under-diagnosis. Universal TcB screening may be a valuable approach to avoid this risk and is currently under investigation in the primary care setting (Netherlands Trial Register: NTR7187).

In the Pakistani study it would have been informative to have other indicators evaluated as well to determine added value and/or safety aspects, such as changes in exchange transfusions, duration of phototherapy, or duration of hospital admission. The mean TSB peak was relatively low in this study population, 9.2 mg/dL before and 10.2 mg/dL after TcB implementation.[5] Most likely these low risk infants are not representative of the large proportion of infants with severe hyperbilirubinemia admitted to non-private, non-tertiary care hospitals in Pakistan. Some other limitations of this study were the small sample size, short duration of the study (12 months) and the single centre design. Whether TcB devices can impact the high incidence of severe hyperbilirubinemia at the population level in low and lower middle-income countries like Pakistan is doubtful. That is, the majority of patients with extreme hyperbilirubinaemia and moderate to severe stages of acute bilirubin encephalopathy present from home. As such, early recognition of clinically relevant hyperbilirubinaemia is particularly needed in primary care/community based settings. Whereas the current study indicates that TcB screening may be useful in a large institution, the price of TcB devices and logistical challenges are obvious barriers to implementation in primary health care settings in low and lower middle income countries. Low-cost and minimally invasive tools for hyperbilirubinaemia recognition including smartphone technology and point-of-care TSB quantification have emerged and may hold promise for early detection of

Confidential: For Review Only

hyperbilirubinaemia in poor-resource settings.[1] Proper education of communities and health care providers remains essential to increase awareness of the main risk factors for severe hyperbilirubinaemia, and facilitate early recognition, timely referral for evaluation and treatment, and adequate followup.

Confidential: For Review Only

Authorship statement

SEM wrote the first manuscript draft. BAvdG and JVB provided input and made revisions. All authors have approved the final manuscript version and agree to be accountable for all aspects of the work in ensuring that questions related to the accuracy or integrity of any part of the work are appropriately investigated and resolved.

Acknowledgments

None

Competing interests and funding

The authors declare no competing interests. BAvdG is funded by efficiency research grants from Erasmus MC (2016-16107) and The Netherlands Organisation for Health Research and Development (ZonMw; 80-84300-98-82020). JVB is supported by personal fellowships from Erasmus MC and The Netherlands Lung Foundation (4.2.14.063JO).

Data sharing, patient consent, ethical approval

Not applicable

Confidential: For Review Only

References

1 Olusanya BO, Kaplan M, Hansen TW. Neonatal hyperbilirubinaemia: a global perspective. *Lancet Child Adolesc Health* 2018;2:P610-20.

2 Shapiro S, Le Pichon JB, Riordan SM, *et al.* The Neurological Sequelae of Neonatal Hyperbilirubinemia: Definitions, diagnosis and treatment of the Kernicterus Spectrum Disorders (KSDs). *Curr Pediatr Rev* 2017;13:199–209.

3 Slusher TM, Zamora TG, Appiah D, *et al.* Burden of severe neonatal jaundice: a systematic review and meta-analysis. *BMJ Paediatr Open* 2017;1:e000105.

4 Wainer S, Parmar SM, Allegro D, *et al.* Impact of a transcutaneous bilirubinometry program on resource utilization and severe hyperbilirubinemia. *Pediatrics* 2012;129:77–86.

5 Shah M, Ariff S, Ali S, *et al*. A quality improvement initiative using transcutaneous bilirubin nomogram to decrease serum bilirubin sampling in low-risk babies. *BMJ Paediatr Open;* in press.

Confidential: For Review Only

BMJ Paediatrics Open

Management of neonatal jaundice in low and lower middle income countries

Journal:	*BMJ Paediatrics Open*
Manuscript ID	bmjpo-2018-000408.R1
Article Type:	Editorial
Date Submitted by the Author:	14-Jan-2019
Complete List of Authors:	Mir, Shahryar; Dijklander Hospital, Paediatrics<br>van der Geest, Berthe; Erasmus MC, Paediatrics<br>Been, Jasper; Erasmus MC, Paediatrics
Keywords:	Comm Child Health, Epidemiology, Jaundice, Neonatology, Screening

SCHOLARONE™
Manuscripts

Management of neonatal jaundice in low and lower middle income countries

Shahryar E. Mir[1], Berthe A.M. van der Geest[2,3], Jasper V. Been[2-4*]

[1]Department of Paediatrics, Dijklander Hospital, Purmerend, the Netherlands

[2]Division of Neonatology, Department of Paediatrics, Erasmus MC – Sophia Children's Hospital, University Medical Centre Rotterdam, Rotterdam, the Netherlands

[3]Department of Obstetrics and Gynaecology, Erasmus MC, University Medical Centre Rotterdam, Rotterdam, the Netherlands

[4]Department of Public Health, Erasmus MC, University Medical Centre Rotterdam, Rotterdam, the Netherlands

[*]Corresponding author: Dr. Jasper V. Been, Division of Neonatology, Department of Paediatrics, Erasmus MC – Sophia Children's Hospital, University Medical Centre Rotterdam, PO Box 2060, 3000 CB Rotterdam, the Netherlands;

E: j.been@erasmusmc.nl; T: +31-10-7037048; F: +31-10-7036815.

Word count: 931

Confidential: For Review Only

More than 85% of newborns develop some degree of jaundice during the first days of life. Often a benign condition, the yellowish discolouration of the skin, sclerae, and mucous membranes in newborn infants is caused by unconjugated hyperbilirubinaemia, which may result from increased production of bilirubin, limited ability to conjugate bilirubin, and/or slow hepatic-enteric clearance of bilirubin.[1] A small proportion of newborns develops extreme hyperbilirubinaemia, i.e. a total serum bilirubin (TSB) level of 25 mg/dL (428 µmol/L) or more. When it is not timely recognised or treated, extreme unconjugated hyperbilirubinaemia may lead to acute bilirubin encephalopathy. This in turn carries a risk of developing a spectrum of long-term neurological sequelae known as kernicterus spectrum disorders (KSD), encompassing “classical kernicterus” but also milder forms of permanent brain damage caused by bilirubin neurotoxicity.[1,2] Worldwide, it is estimated that extreme hyperbilirubinaemia affects at least 481,000 late-preterm and term newborn infants annually, resulting in 114,000 deaths and more than 63,000 survivors with moderate or severe long-term disability.[1,3] More than 75% of affected infants live in low and lower middle-income countries. Moreover, in South Asia severe hyperbilirubinaemia is the seventh leading cause of neonatal mortality.[1]

When timely recognised, clinically significant hyperbilirubinaemia can be treated with conventional phototherapy and in more severe cases exchange transfusion, thus reducing the risk of KSD. Relatively uncommon in high-income countries, exchange transfusions are performed regularly in low and lower middle-income countries.[3] In these countries early recognition of hyperbilirubinaemia can be difficult. In low-resource settings home births are common, and for institutional deliveries early post-discharge follow-up controls are not always feasible. In addition, parental unawareness and logistical challenges may result in poor care-seeking behaviour. In these settings infants often present with extreme hyperbilirubinemia and moderate to severe stages of acute bilirubin encephalopathy, resulting in high mortality and morbidity rates.[1,3] Traditionally, neonatal jaundice is determined by visual inspection. If severe jaundice is suspected, TSB is quantified in blood to assess the need for treatment. However, visual inspection of neonatal jaundice correlates poorly with the TSB level, particularly in non-Caucasian infants.[1] Moreover, in areas with limited resources laboratory testing can be challenging due to the high cost and lack of trained technical personnel. Transcutaneous bilirubin (TcB) devices have been developed as potential non-invasive alternatives to visual inspection. Several observational studies have shown a good correlation between TSB and TcB measurements. TcB can be performed by nurses or trained health workers with limited education. Coordinated TcB screening in a Canadian community setting was associated with a 55% reduction in severe hyperbilirubinaemia compared with visual inspection alone.[4]

Confidential: For Review Only

Shah *et al.* have evaluated a care improvement initiative in a large hospital in Karachi, the largest city in Pakistan.[5] TcB screening of visibly jaundiced, otherwise well babies was implemented and clinical characteristics and jaundice-related outcomes were compared in the six months before versus the six months after implementation. There was a 79% reduction in the number of blood samples taken for TSB quantification following TcB implementation; at the same time the proportion of infants receiving phototherapy increased (6% after vs. 4% before implementation of TcB). Overall this is a useful uncontrolled before-after study, and the first to investigate the effectiveness of using a TcB device to diagnose neonatal jaundice in a Pakistani population. The considerable reduction in TSB sampling following TcB implementation as compared to other studies (in general reduction of 40 to 50%), was attributed to excessive sampling due to the high incidence of severe hyperbilirubinaemia (15%) in South Asia. The increased use of phototherapy in the post-implementation period suggests improved recognition of hyperbilirubinaemia, although it is possible that this includes some over-treatment. In this study the vast majority of infants were indeed screened, suggesting a low threshold for selection for screening. It is however important to note that selective screening based on visible jaundice carries a risk of under-diagnosis. Universal TcB screening may be a valuable approach to avoid this risk and is currently under investigation in the primary care setting (Netherlands Trial Register: NTR7187).

In the Pakistani study it would have been informative to have other indicators evaluated as well to determine added value and/or safety aspects, such as changes in exchange transfusions, duration of phototherapy, or duration of hospital admission. The mean TSB peak was relatively low in this study population, 9.2 mg/dL before and 10.2 mg/dL after TcB implementation.[5] Most likely these low risk infants are not representative of the large proportion of infants with severe hyperbilirubinemia admitted to non-private, non-tertiary care hospitals in Pakistan. Some other limitations of this study were the small sample size, short duration of the study (12 months) and the single centre design. Whether TcB devices can impact the high incidence of severe hyperbilirubinemia at the population level in low and lower middle-income countries like Pakistan is doubtful. That is, the majority of patients with extreme hyperbilirubinaemia and moderate to severe stages of acute bilirubin encephalopathy present from home. As such, early recognition of clinically relevant hyperbilirubinaemia is particularly needed in primary care/community based settings. Whereas the current study indicates that TcB screening may be useful in a large institution, the price of TcB devices and logistical challenges are obvious barriers to implementation in primary health care settings in low and lower middle income countries. Low-cost and minimally invasive tools for hyperbilirubinaemia recognition including smartphone technology and point-of-care TSB quantification have emerged and may hold promise for early detection of

Confidential: For Review Only

hyperbilirubinaemia in poor-resource settings.[1] Proper education of communities and health care providers remains essential to increase awareness of the main risk factors for severe hyperbilirubinaemia, and facilitate early recognition, timely referral for evaluation and treatment, and adequate followup.

Confidential: For Review Only

Authorship statement

SEM wrote the first manuscript draft. BAvdG and JVB provided input and made revisions. All authors have approved the final manuscript version and agree to be accountable for all aspects of the work in ensuring that questions related to the accuracy or integrity of any part of the work are appropriately investigated and resolved.

Acknowledgments

None

Competing interests and funding

The authors declare no competing interests. SEM is supported by the AMAYA Initiative. BAvdG is funded by efficiency research grants from Erasmus MC (2016-16107) and The Netherlands Organisation for Health Research and Development (ZonMw; 80-84300-98-82020). JVB is supported by personal fellowships from Erasmus MC and The Netherlands Lung Foundation (4.2.14.063JO).

Data sharing, patient consent, ethical approval

Not applicable

Confidential: For Review Only

References

1 Olusanya BO, Kaplan M, Hansen TW. Neonatal hyperbilirubinaemia: a global perspective. *Lancet Child Adolesc Health* 2018;2:P610-20.

2 Shapiro S, Le Pichon JB, Riordan SM, *et al.* The Neurological Sequelae of Neonatal Hyperbilirubinemia: Definitions, diagnosis and treatment of the Kernicterus Spectrum Disorders (KSDs). *Curr Pediatr Rev* 2017;13:199–209.

3 Slusher TM, Zamora TG, Appiah D, *et al.* Burden of severe neonatal jaundice: a systematic review and meta-analysis. *BMJ Paediatr Open* 2017;1:e000105.

4 Wainer S, Parmar SM, Allegro D, *et al.* Impact of a transcutaneous bilirubinometry program on resource utilization and severe hyperbilirubinemia. *Pediatrics* 2012;129:77–86.

5 Shah M, Ariff S, Ali S, *et al*. A quality improvement initiative using transcutaneous bilirubin nomogram to decrease serum bilirubin sampling in low-risk babies. *BMJ Paediatr Open;* in press.

Confidential: For Review Only
