# Supplementary material for: Management of neonatal jaundice in low- and lower-middle-income countries
Source: BMJ Paediatr Open. 2019 Feb 14;3(1):e000408. doi: 10.1136/bmjpo-2018-000408 (PMC6422246; doi:10.1136/bmjpo-2018-000408)
Supplement: Reviewer comments [file bmjpo-2018-000408.reviewer_comments.pdf]

PEER REVIEW HISTORY

BMJ Paediatrics Open publishes all reviews undertaken for accepted manuscripts. Reviewers are asked to complete a checklist review form and are provided with free text boxes to elaborate on their assessment. These free text comments are reproduced below.

ARTICLE DETAILS

**TITLE (PROVISIONAL)**	Management of neonatal jaundice in low and lower middle income countries
**AUTHORS**	Been, Jasper; Mir, Shahryar; van der Geest, Berthe

VERSION 1 – REVIEW

**REVIEWER**	Reviewer name: Allegaert karel<br>Institution and Country: ku leuven, Belgium and Erasmus mc Rotterdam, the netherlands<br>Competing interests: I was the handling editor of the original paper that initiated this editorial, and work in the same institute as the authors of the editorial
**REVIEW RETURNED**	07-Jan-2019

**GENERAL COMMENTS**	I have carefully read this editorial, and have enjoyed its reading, well balanced paper, and also focus on the add on and limitations of the original paper. I suggest to accept this version

**REVIEWER**	Reviewer name: Peter Rohloff<br>Institution and Country: Brigham and Women's Hospital USA, Maya Health Alliance Guatemala<br>Competing interests: Associate Editor, BMJ Paeds Open
**REVIEW RETURNED**	12-Jan-2019

**GENERAL COMMENTS**	This is an excellent commentary providing a good framing and a balanced discussion of the strengths and weakness of the article in question.
